# Long non-coding RNA MCM3AP-AS1 promotes growth and migration through modulating FOXK1 by sponging miR-138-5p in pancreatic cancer

**DOI:** 10.1186/s10020-019-0121-2

**Published:** 2019-12-12

**Authors:** Ming Yang, Shijuan Sun, Yao Guo, Junjie Qin, Guangming Liu

**Affiliations:** 1 Department of Pancreatic Surgery, Union Hospital, Tongji Medical College, Huazhong University of Science and Technology, Wuhan City, 430022 Hubei Province China; 2 Department of Gastroenterology, The First Hospital of Jilin University, 71 Xinmin Street, Changchun City, 130021 Jilin Province China

**Keywords:** MCM3AP-AS1, miR-138-5p, Growth, Migration, Pancreatic cancer

## Abstract

**Background:**

Pancreatic cancer (PC) is a type of malignant gastrointestinal tumor. Long non-coding RNA MCM3AP antisense RNA 1 (MCM3AP-AS1) has been reported to stimulate proliferation, migration and invasion in several types of tumors. However, the role of MCM3AP-AS1 in PC remains unclear.

**Methods:**

MCM3AP-AS1, microRNA miR-138-5p (miR-138-5p) and FOXK1 levels were detected using quantitative real time PCR. Cell proliferation, migration and invasion were analyzed. Dual luciferase reporter assay was used to confirm the relationship between MCM3AP-AS1 and miR-138-5p, between miR-138-5p and FOXK1. Protein levels were identified using western blot analysis.

**Results:**

MCM3AP-AS1 overexpression promoted proliferation, migration and invasion in PC cells. MCM3AP-AS1 silencing showed a suppressive effect on cell growth in PC cells. Moreover, MCM3AP-AS1 knockdown suppressed tumor growth in mice. Dual luciferase reporter assay demonstrated MCM3AP-AS1 could sponge microRNA-138-5p (miR-138-5p), and FOXK1 could bind with miR-138-5p. Positive correlation between MCM3AP-AS1 and FOXK1 was testified, as well as negative correlation between miR-138-5p and FOXK1. MCM3AP-AS1 promoted FOXK1 expression by targeting miR-138-5p, and MCM3AP-AS1 facilitated growth and invasion in PC cells by FOXK1.

**Conclusion:**

MCM3AP-AS1 promoted growth and migration through modulating miR-138-5p/FOXK1 axis in PC, providing insights into MCM3AP-AS1/miR-138-5p/FOXK1 axis as novel candidates for PC therapy from bench to clinic.

## Background

Pancreatic cancer (PC) is a type of malignant gastrointestinal tumor, which is characterized by rapid progression, insidious onset and low survival rate (Sun and Zhang 2019; Xia et al. 2019). American Cancer Society estimates the 1-year relative survival rate of PC is about 20%, and 5-year survival rate is approximately 7% (Jemal et al. 2008; Cardenes et al. 2006). Up to date, although there are several therapeutic strategies have been widely utilized, such as radiotherapy, chemotherapy as well as surgical resection, the incidence and mortality of PC are dismal (Heestand and Kurzrock 2015). Therefore, it is urgent to develop novel treatment strategies targeting PC, as well as to investigate the underlying pathogenic mechanisms.

Long non-coding RNAs (lncRNAs) are non-coding RNAs over 200 nucleotides, which regulate gene expression at epigenetic and post-transcriptional levels (Sun and Zhang 2019; Ponting et al. 2009). More and more evidences have reported lncRNAs are associated with tumor pathogenesis, including PC. LINC00339 level was high in PC tissues and cell models. In detail, LINC00339 was reported to enhance cell proliferation and metastasis of PC (Zhang et al. 2019a). Linc-ROR was increased in PC tissues, and ectopic expression of linc-ROR facilitated proliferation, migration and invasion in PC (Zhan et al. 2016). In addition, MCM3AP, which is located at human chromosome 21, is reported to act as a major regulator in DNA replication by acetylating micro-chromosome maintenance protein 3 (MCM3). MCM3AP play an important role in suppressing DNA replication by inhibiting S phase of cell cycle and regulating gene expression in human tumors (Yang et al. 2017; Kuwahara et al. 2016). MCM3AP-AS1 is a lncRNA antisense targeting human MCM3AP gene, which participates in cancer development. Previously, MCM3AP-AS1 was up-regulated in hepatocellular carcinoma tissues, as well as promoted growth of hepatocellular carcinoma (Wang et al. 2019). MCM3AP-AS1 was also found to be elevated in papillary thyroid cancer, and high MCM3AP-AS1 expression stimulated proliferation, migration as well as invasion of papillary thyroid cancer (Liang et al. 2019a). Collectively, we speculated MCM3AP-AS1 regulated the progression of tumors. However, the precise function of MCM3AP-AS1 in PC remains unclear.

MicroRNAs (miRNAs), less than 25 nucleotides, are related with various biological processes (Barbato et al. 2017; Hu et al. 2019). Yu et al. discovered that miR-138-5p exhibited a suppressive effect on development of PC, leading to suppression of tumor formation (Yu et al. 2015). Tian and colleagues reported miR-138-5p repressed autography as well as tumor growth in PC (Tian et al. 2017), indicating miR-138-5p might act an important role in PC. Importantly, lncRNAs could participated in regulation of tumors (Barbato et al. 2017) by sponging miRNAs. Hence, we hypothesized that MCM3AP-AS1 might participate in progression of PC via targeting miR-138-5p. With the aim to explore novel candidates for PC therapeutics, effects of MCM3AP-AS1 on cell proliferation, migration as well as invasion of PC was investigated in cell models, and its role in PC was further validated in vivo.

## Methods

### Tissue samples

86 pairs of PC tissues and adjacent normal tissues were harvested from Union Hospital, Tongji Medical College, Huazhong University of Science and Technology. Detailed characteristics of patients were presented in Table 1. All patients were given the written informed consent. After surgical resection treatments, PC tissues were immediately stored in liquid nitrogen. This study was carried out according to Declaration of Helsinki and approved by Ethical Committee for Union Hospital Affiliated with Tongji Medical College of Huazhong University of Science and Technology.

**Table 1:** The characteristics of the patients with PC

Characteristics	Number of patients	lncRNA MCM3AP-AS1 expression		*P* value
		Low (<median)	High (≥median)	
Number	86	43	43	
Gender				
Male	39	20	19	0.829
Female	47	23	24	
Age (years)				
< 60	33	16	17	0.825
≥ 60	53	27	26	
Tumor size (cm)				
< 2	44	28	16	0.010*
≥ 2	42	15	27	
TNM stage				
I-II	50	30	20	0.029*
III-IV	36	13	23	
Tumor differentiation				
Well/Moderate	35	20	15	0.272
Poor	51	23	28	
Lymph node metastasis				
Negative	50	32	18	0.002*
Positive	36	11	25	
Distant metastasis				
Negative	46	26	20	0.195
Positive	40	17	23	

### Cell culture and transfection

A pancreatic duct epithelial cell line (HPDE6-C7), human PC cell lines (PANC-1, BxPC-3, MIA PaCa-2, Capan-2, AsPC-1) as well as HEK-293 cell line were purchased from Beijing Zhongyuan Ltd. (China). Cells were maintained in DMEM containing 10% fetal bovine serum (FBS) at 37 °C in 5% CO_2_. PANC-1 as well as AsPC-1 cells were transfected to overexpress MCM3AP-AS1 in vitro, whereas BxPC-3 as well as Capan-2 cells were transfected to silence MCM3AP-AS1.

### In situ hybridization (ISH)

Tissues were cut into 5 μm and dewaxed. Sections were treated with 20 μg/mL protease K at 37 °C for 10 min. ISH buffer was used to pre-hybridize the sample and then sections were incubated with digoxigenin-labelled probe for 40 min at 45 °C. Then, digoxigenin antibody was incubated with sections overnight 4 °C. Finally, nitroblue tetrazole/5-bromo-4-chloro-3-indolyl phosphate was applied and images were captured using a microscope.

### Quantitative real time PCR

Total RNAs were isolated through TRIzol reagent (Beyotime, Shanghia, China). RNA was reversely transcribed into cDNA. RNA level of MCM3AP-AS1, miR-138-5p and FOXK1 was examined using SYBR Green (Solarbio, Beijing, China). Relative gene expression was normalized to GAPDH or U6 using 2^-ΔΔCt^ method. Primers were listed in Table 2.

**Table 2:** The primers were used in this study

Name	Sequence (5′-3′)
MCM3AP-AS1	F: GCTGCTAATGGCAACACTGA
	R: AGGTGCTGTCTGGTGGAGAT
miR-138-5p	F: AGCTGGTGTTGTGAATCAGGCCG
	R: AACGCTTCACGAATTTGCGT
FOXK1	F: GCCTCCTTGACAATACCGCT
	R: TTCCAAACCCTCCCTCTGGT
GAPDH	F: CAGGAGGCATTGCTGATGAT
	R: GAAGGCTGGGGCTCATTT
U6	F: CTCGCTTCGGCAGCACA
	R: AACGCTTCACGAATTTGCGT

### MTT assay

Cells (3*10^3^) were plated in 96-well plates. 24 h post-seeding, 10 μL of the MTT solution (Beyotime, Shanghia, China) were added into cells and incubated for 5 h. The absorbance at 570 nm was measured with a microplate reader.

### Cell migration and invasion assay

To detect cell migration ability, PANC-1 and AsPC-1 cells were seeded in 6-well plates. A straight scratch was made by the pipette tip, and then the width of the wounding scratches was measured. Images were photographed at 0 h and 24 h under a microscope. For cell invasion analysis, PANC-1 and AsPC-1 cells (3*10^4^ per well) were resuspended in 200 μL serum-free medium in the upper chamber coated with Matrigel. 800 μL of medium containing 10% FBS was added into the lower chamber. 48 h post-seeding, cells were fixed ad stained with violet crystalline (Takara, Dalian, China).

### Colony formation assay

PANC-1 and AsPC-1 cells (1*10^3^) were seeded in a 6-well plate. Cells were cultured for 2 weeks. After fixation with 4% paraformaldehyde for 25 min, cells were stained with 0.5% crystal violet for 25 min. Images were photographed under a microscope.

### Western blot analysis

Total proteins were extracted via RIPA buffer and quantified using a BCA assay kit (Solarbio, Beijing, China). 40 μg sample were separated by SDS-PAGE and transferred onto PVDF membranes. Subsequently, the membranes were blocked with 5% non-fat milk for 50 min at 37 °C, and covered with primary antibodies overnight at 4 °C. The primary antibodies were as follows: PCNA (1:1000), p21 (1:1000), MMP2 (1:1000), MMP9 (1:1000) and GAPDH (1:10000), (all from Bioworld, Minneapolis, MN, USA). Afterwards, HRP secondary antibodies (1:3000, SCBT, Santa Cruz, CA, USA) were used to incubate with membrane for 1 h at 37 °C. Protein bands were visualized through ECL detection kit (Takara, Dalian, China). GAPDH was utilized as an internal control.

### Dual luciferase reporter assay

The partial sequences of MCM3AP-AS1 containing miR-138-5p binding site or losing miR-138-5p binding site, named by (MCM3AP-AS1-WT or MCM3AP-AS1-MUT), were inserted into pmirGLO plasmids (Beyotime, Shanghia, China). Moreover, the wild-type 3′-UTR and the mutant 3′-UTR of FOXK1, named FOXK1-WT and FOXK1-MUT, were cloned into pmirGLO plasmids. HEK-293 cells were co-transfected with MCM3AP-AS1 pmirGLO plasmids (MCM3AP-AS1-WT or MCM3AP-AS1-MUT) and miRNAs (miR-NC or miR-138-5p), as well as FOXK1 pmirGLO plasmids (FOXK1-WT or FOXK1-MUT) and miRNAs (miR-NC or miR-138-5p). 48 h post-transfection, dual-luciferase reporter assay system (Takara, Dalian, China) was used to examine relative luciferase activities.

### RNA immunoprecipitation (RIP)

Cells were collected and lysed using lysis buffer. The supernatant from cell lysates was incubated with human anti-Ago2 antibody (SCBT, Santa Cruz, CA, USA) or negative control antibody (mouse IgG, SCBT, Santa Cruz, CA, USA) for 4 h. Proteins were digested via Proteinase K buffer and the quantitative real time PCR examined co-precipitated RNAs. Total RNAs were regarded as Input control.

### Tumor xenograft model

4~5-week-old BALB/c nude mice (18–20 g) were purchased from Vital River Laboratory Animal Technology Ltd. (Beijing, China). All experiments were performed according to Guideline for the Care and Use of Laboratory Animals and approved by Ethical Committee for Union Hospital Affiliated with Tongji Medical College of Huazhong University of Science and Technology. Mice were divided into two groups (*n* = 12/group): (i) shRNA-NC; (ii) MCM3AP-AS1 shRNA-2# (shRNA-2#). BxPC-3 cells (1*10^6^) expressing shRNA-NC or shRNA-2# were subcutaneously injected to the right flank of the mice. The tumor volume was examined every 5 days and calculated with the formula: V = (length × width^2^)/2. The tumor weight was also weighed and recorded. Thirty days post-transplantation, all mice were sacrificed and tumor tissues were collected.

### Statistics

Data analysis was presented as means ± SD and analyzed through SPSS 23.0. Survival of PC was compared with the Mann-Whitney U test. The t-test was used to assay significance of difference between two groups. One-way of ANOVA by Tukey’s test was carried to analyze multiple comparisons. *P*-value ≤ 0.05 was considered statistically significant.

## Results

### MCM3AP-AS1 was up-regulated in PC tissues and cell models

**Fig. 1:**
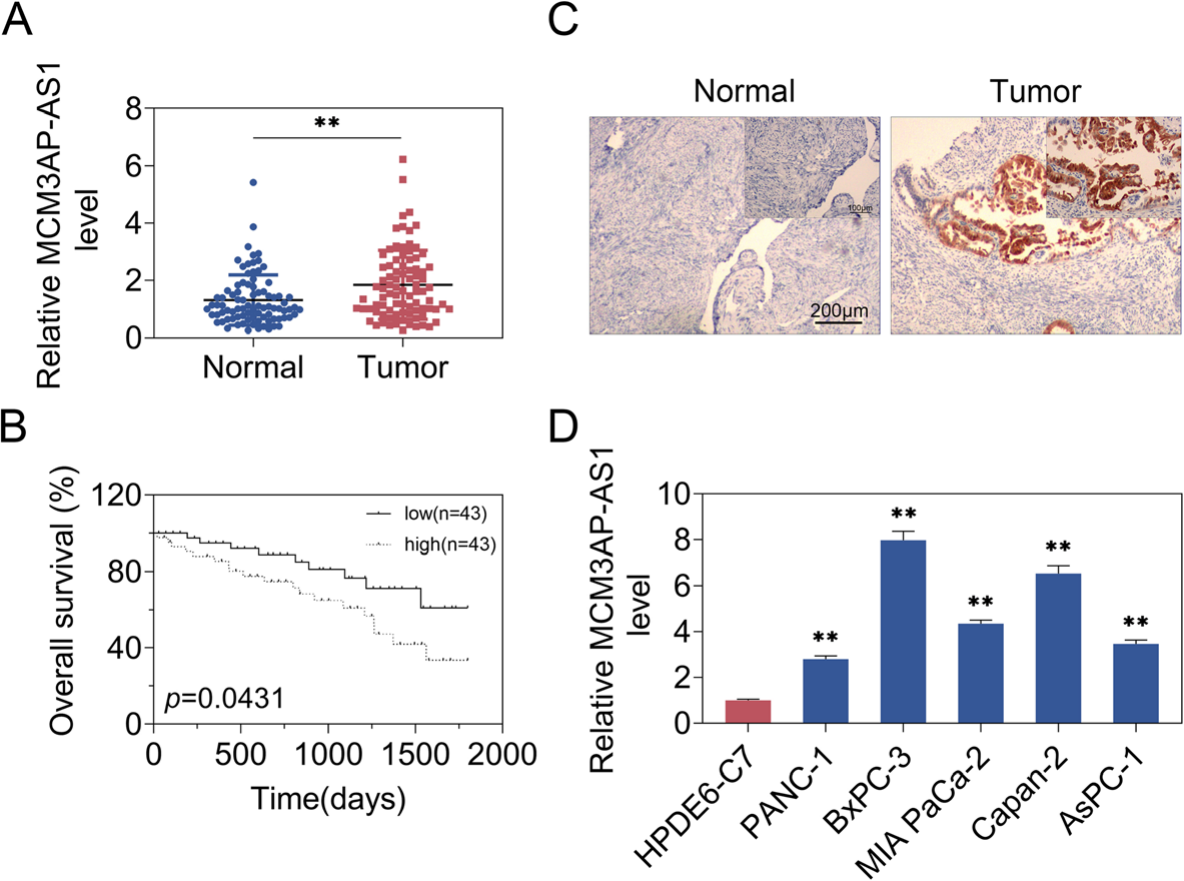
MCM3AP-AS1 was up-regulated in PC. **a** qRT-PCR measured MCM3AP-AS1 level in n PC tissues and adjacent normal tissues. *n* = 86. **b** Overall survival was analyzed. n = 86. **c** ISH detected MCM3AP-AS1 expression. Scale bar, 50 μm. **d** MCM3AP-AS1 level was examined using quantitative real time PCR in PC cell lines and pancreatic duct epithelial cells. *n* = 3. (**, *p* < 0.01)

qRT-PCR results showed that MCM3AP-AS1 level was enhanced in PC tissues compared with that in adjacent normal tissues (Fig. 1a). Moreover, we found that the patients with high MCM3AP-AS1 level exhibited shorter survival rates (Fig. 1b). ISH verified that MCM3AP-AS1 was also elevated in PC tissues (Fig. 1c). qRT-PCR analysis further demonstrated that MCM3AP-AS1 level was higher in PC cell models (PANC-1, BxPC-3, MIA PaCa-2, Capan-2, AsPC-1) compared to that in pancreatic duct epithelial cell line (HPDE6-C7) (Fig. 1d). To sum up, these data indicated MCM3AP-AS1 was increased in context of PC.

### MCM3AP-AS1 promoted growth and invasion in PC cell models

**Fig. 2:**
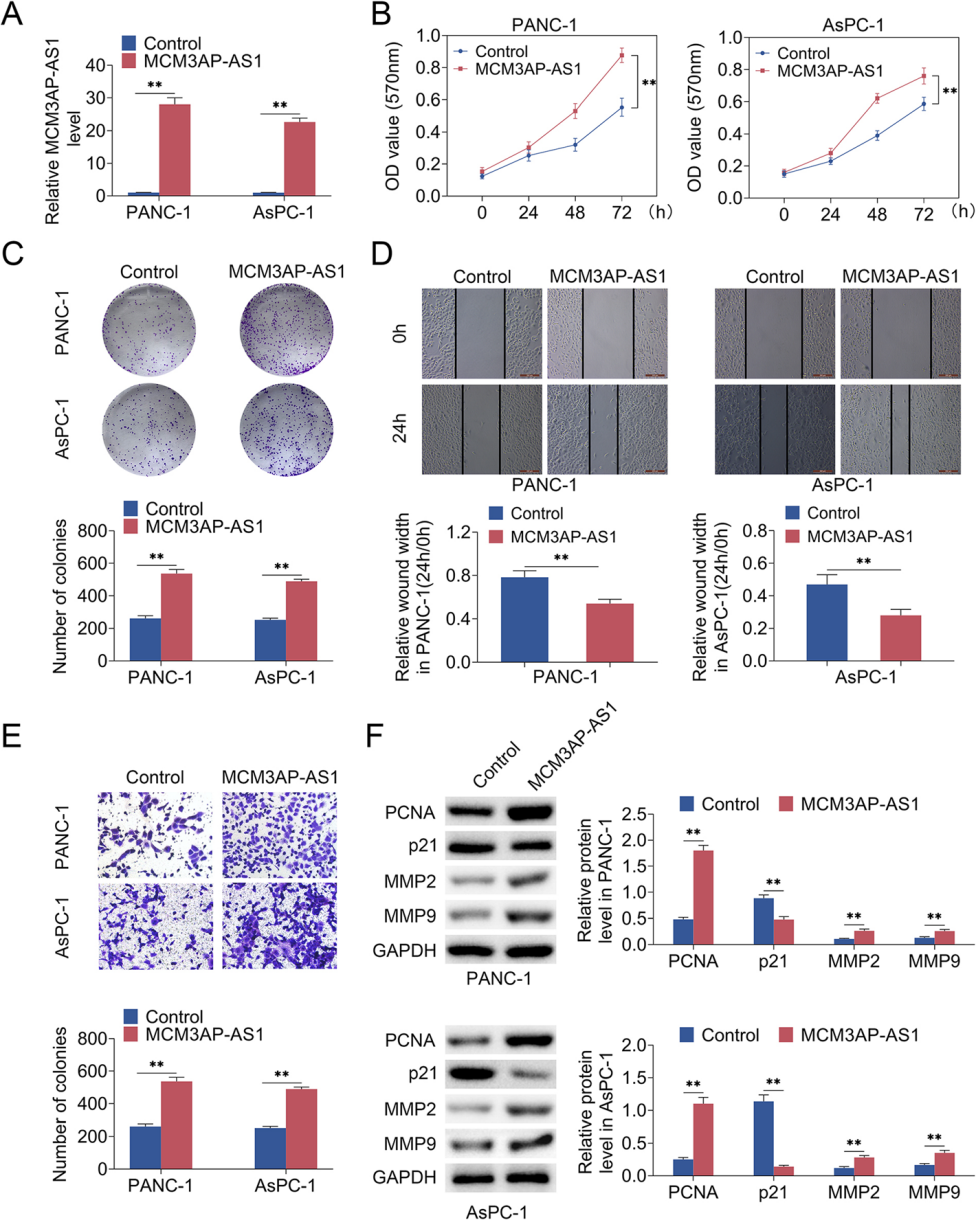
MCM3AP-AS1 promoted cell growth and invasion in PC cells. **a** qRT-PCR identified MCM3AP-AS1 level. **b** MTT assay detected cell proliferation. **c** Clone formation ability was detected using clone formation assay. **d**, **e** Transwell assay and wound healing assay examined cell invasion and migration. Scale bar: 50 μm. **f** PCNA, MMP2, MMP9 and p21 protein levels were examined using western blot analysis. *n* = 3. (**, *p* < 0.01)

Next, effects of MCM3AP-AS1 on growth and invasion in PC cells was investigated. As shown in Fig. 1d, we found MCM3AP-AS1 level was decreased in PANC-1 and AsPC-1 cell lines. To rescue MCM3AP-AS1 level, MCM3AP-AS1 was transiently overexpressed in PANC-1 and AsPC-1 cells using lipofectmine. qRT-PCR showed MCM3AP-AS1 level was restored in MCM3AP-AS1-overexpression cells (Fig. 2a). MTT assay suggested MCM3AP-AS1 overexpression promoted PC cell proliferation (Fig. 2b) as well as clone formation ability (Fig. 2c). Transwell assay and wound healing assay revealed that cell invasion and migration abilities were elevated upon MCM3AP-AS1 overexpression (Fig. 2d and e). Increased protein levels of PCNA, MMP2 and MMP9 and decreased p21 level were detected under MCM3AP-AS1 overexpression using western blot analysis (Fig. 2f). These findings suggested MCM3AP-AS1 promoted growth as well as invasion in PC cell models.

### MCM3AP-AS1 knockdown inhibited growth and invasion in PC cell models, and suppressed tumor growth in mice

**Fig. 3:**
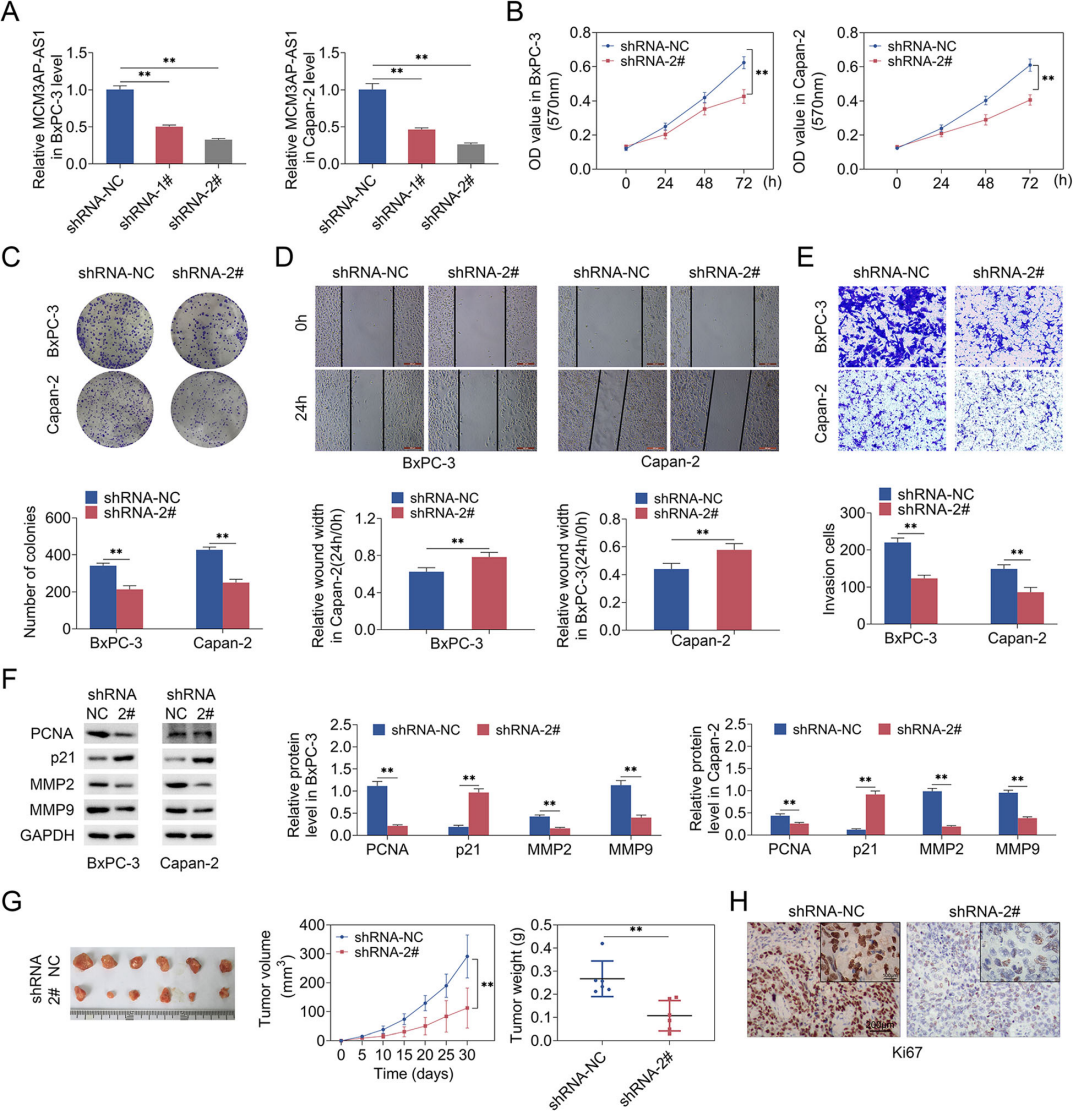
MCM3AP-AS1 knockdown inhibited cell growth and invasion in PC cells, and suppressed tumor growth in mice. **a** qRT-PCR was used to measure MCM3AP-AS1 level. **b** Cell proliferation was detected using MTT assay. **c** Clone formation assay was used to measure clone formation ability. **d**-**e** Cell invasion and migration was examined by Transwell assay and wound healing assay. Scale bar: 50 μm. **f** Western blot analysis detected PCNA, MMP2, MMP9 and p21 protein levels. n = 3. **g** Tumor volume and weight were examined. **h** Immunohistochemical staining testified Ki67 expression. Scale bar: 50 μm. (**, *p* < 0.01)

We then investigated role of MCM3AP-AS1 in cell growth as well as tumor growth. In Fig. 1d, MCM3AP-AS1 level was relatively high in BxPC-3 and Capan-2 cells, therefore, the MCM3AP-AS1 silencing was performed those two cell lines. In vitro, qRT-PCR assay validated the efficiency of MCM3AP-AS1 silencing (Fig. 3a). Additionally, MCM3AP-AS1 silencing repressed PC cell proliferation (Fig. 3b), and clone formation ability (Fig. 3c). Cell invasion and migration was reduced upon MCM3AP-AS1 silencing, evidenced by Transwell and wound healing assay (Fig. 3d and e). Western blot analysis demonstrated that MCM3AP-AS1 silencing down-regulated PCNA, MMP2 and MMP9 protein levels and enhanced p21 protein level in BxPC-3 and Capan-2 cells. Furthermore, MCM3AP-AS1 knockdown repressed tumor growth in mice, including decreased tumor volume and weight (Fig. 3g). Immunohistochemical staining showed MCM3AP-AS1 knockdown reduced Ki67 expression (Fig. 3h). To sum up, these results suggested MCM3AP-AS1 knockdown repressed growth and invasion in vitro, and suppressed tumor growth in vivo.

### MCM3AP-AS1 was negatively associated with miR-138-5p

**Fig. 4:**
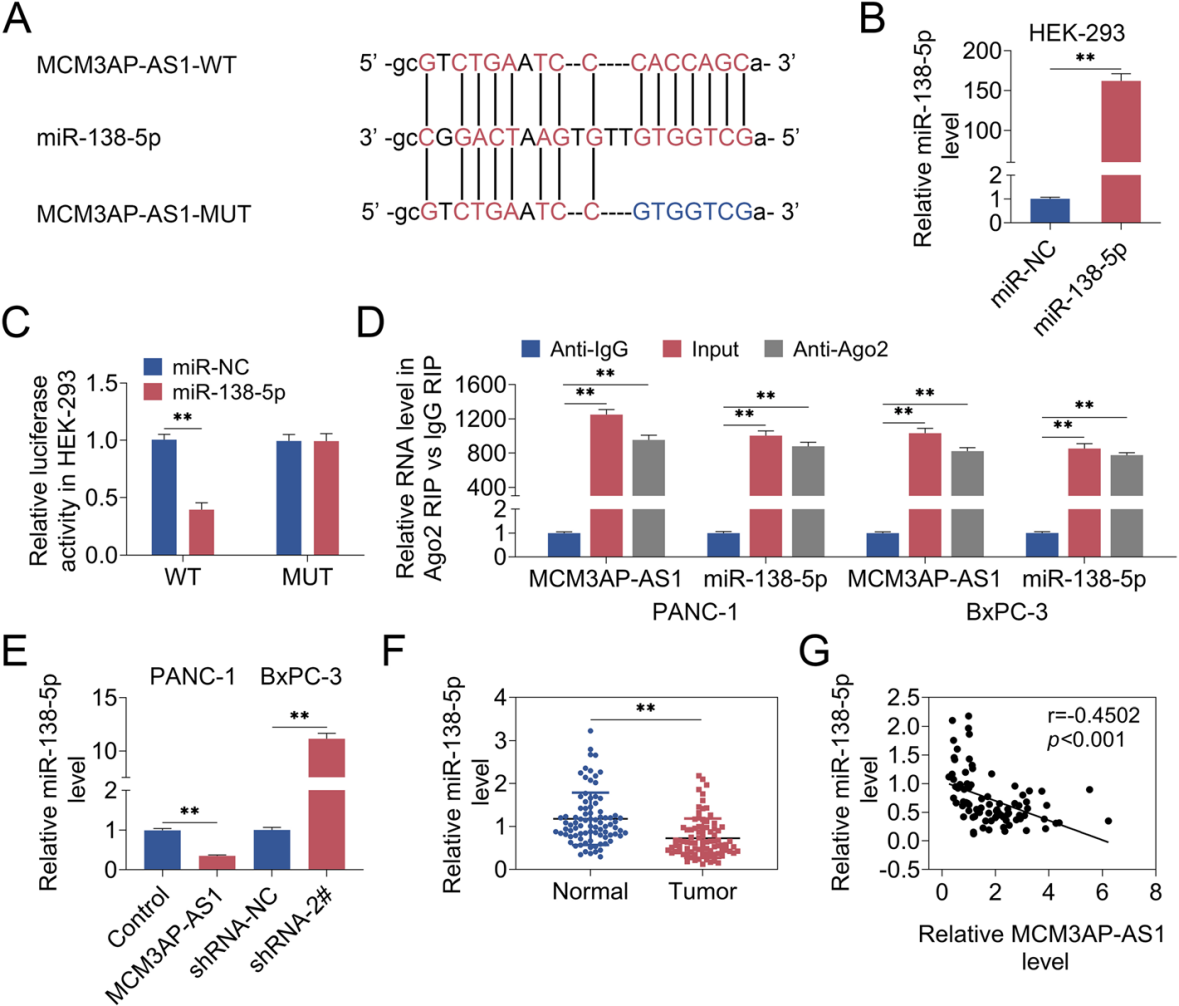
MCM3AP-AS1 was negatively associated with miR-138-5p. **a** Bioinformatic analysis predicted that MCM3AP-AS1 could sponge miR-138-5p. **b** qRT-PCR detected miR-138-5p level. **c** Dual luciferase reporter assay demonstrated the interaction between MCM3AP-AS1 and miR-138-5p. **d** The binding of MCM3AP-AS1 and miR-138-5p was identified via RIP assay. **e**-**f** qRT-PCR analyzed miR-138-5p level. **g** The correlation between MCM3AP-AS1 and miR-138-5p was assayed. n = 3. (**, *p* < 0.01)

To explore the mechanisms of MCM3AP-AS1-mediated PC progression, bioinformatic analysis was performed. Interestingly, bioinformatic analysis predicted MCM3AP-AS1 could sponge miR-138-5p (Fig. 4a). qRT-PCR proved that miR-138-5p level was higher in HEK-293 cells expressing miR-138-5p mimics than those expressing miR-NC (Fig. 4b). Dual luciferase reporter assay testified that miR-138-5p reduced the luciferase activity in HEK-293 cells co-expressing MCM3AP-AS1-WT, whereas there were no effects on HEK-293 cells co-expressing miR-138-5p and MCM3AP-AS1-MUT (Fig. 4c). Further, RIP assay revealed that MCM3AP-AS1 and miR-138-5p levels were enriched in Ago2 pellet compared with those in IgG pellet, as well as compared with cell lysates (Input) (Fig. 4d). Then, MCM3AP-AS1 was overexpressed in PANC-1 cells and silenced in BxPC-3 cells. qRT-PCR assay illustrated miR-138-5p level was decreased upon MCM3AP-AS1 overexpression, which could be restored by MCM3AP-AS1-silencing (Fig. 4e). Moreover, miR-138-5p level was reduced in PC tissues compared with that in adjacent normal tissues (Fig. 4f). There was a negative correlation existed between MCM3AP-AS1 and miR-138-5p (Fig. 4g). Combined, these results illustrated MCM3AP-AS1 was negatively associated with miR-138-5p.

### MCM3AP-AS1 promoted FOXK1 expression by targeting miR-138-5p

**Fig. 5:**
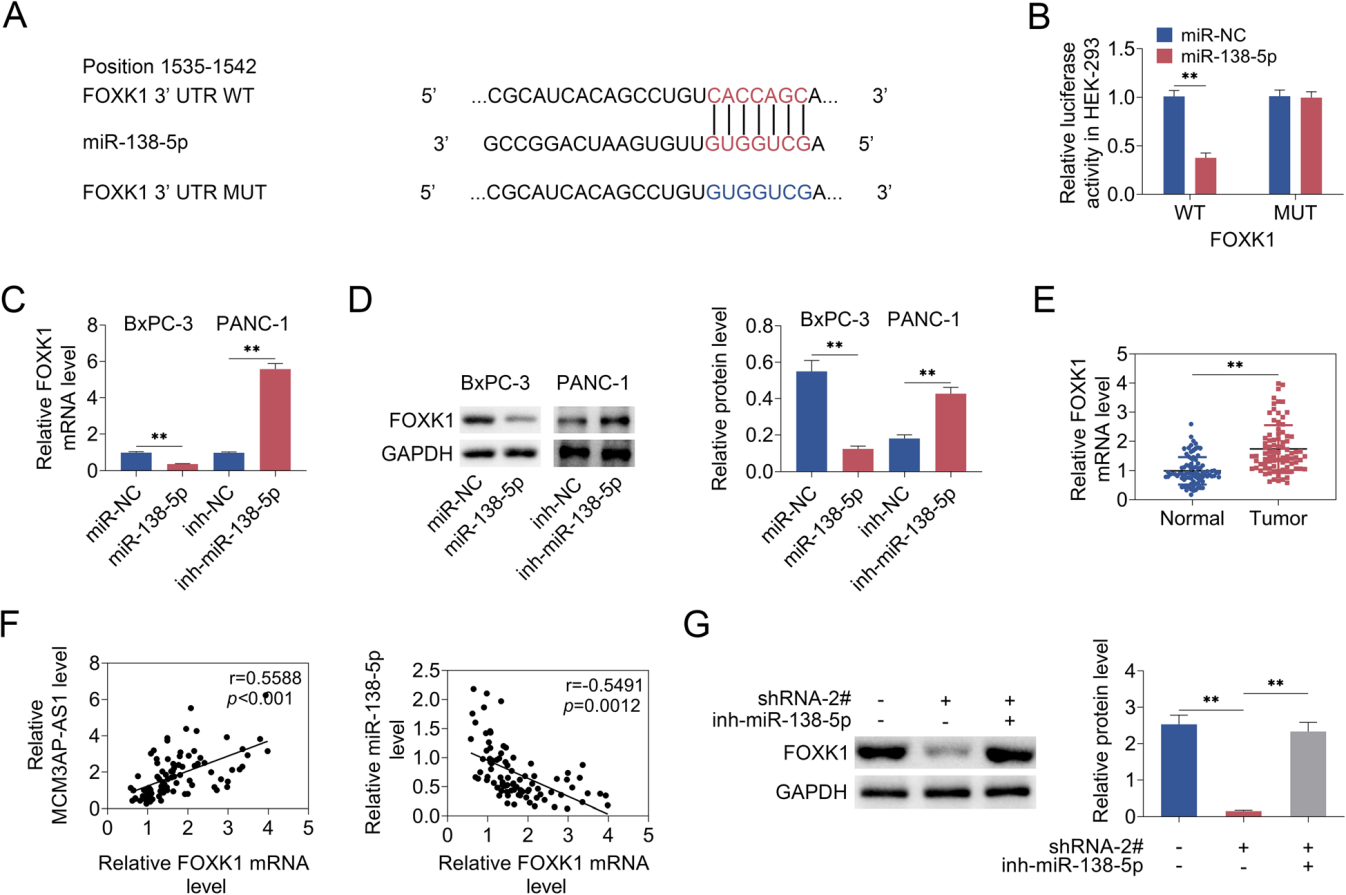
MCM3AP-AS1 promoted FOXK1 expression by targeting miR-138-5p. **a** Bioinformatic analysis predicted that miR-138-5p could target FOXK1. **b** Dual luciferase reporter assay confirmed the relationship between miR-138-5p and FOXK1. **c** qRT-PCR detected miR-138-5p level. **d**-**f** qRT-PCR and western blot analysis measured FOXK1 level. **g** The correlation between miR-138-5p and FOXK1 was identified. (H) FOXK1 level was examined using quantitative real time PCR and western blot analysis. n = 3. (**, *p* < 0.01)

To validate the bioinformatic predictions (Fig. 5a), dual luciferase reporter assay was performed and identified that the luciferase activity in HEK-293 cells co-expressing miR-138-5p mimics and FOXK1-WT was reduced. However, the luciferase activity in HEK-293 cells co-expressing miR-138-5p and FOXK1-MUT exhibited no effects (Fig. 5b). To detect FOXK1 expression, qRT-PCR and western blot analysis revealed that FOXK1 expression was decreased in BxPC-3 cells upon miR-138-5p mimics, which was elevated in PANC-1 cells expressing miR-138-5p inhibitor (Fig. 5c and d). Moreover, FOXK1 level was up-regulated in PC tissues (Fig. 5e). MCM3AP-AS1 and FOXK1 levels showed positive correlation, whereas miR-138-5p and FOXK1 levels exhibited negative correlation (Fig. 5f). FOXK1 protein expression was inhibited in MCM3AP-AS1-silencing cells, whereas which was reversed in BxPC-3 cells co-expressing MCM3AP-AS1-silencing and miR-138-5p inhibitor (Fig. 5g). These findings implied that MCM3AP-AS1 promoted FOXK1 expression by targeting miR-138-5p.

### MCM3AP-AS1 facilitated cell growth and invasion in PC cells by regulating FOXK1 expression

**Fig. 6:**
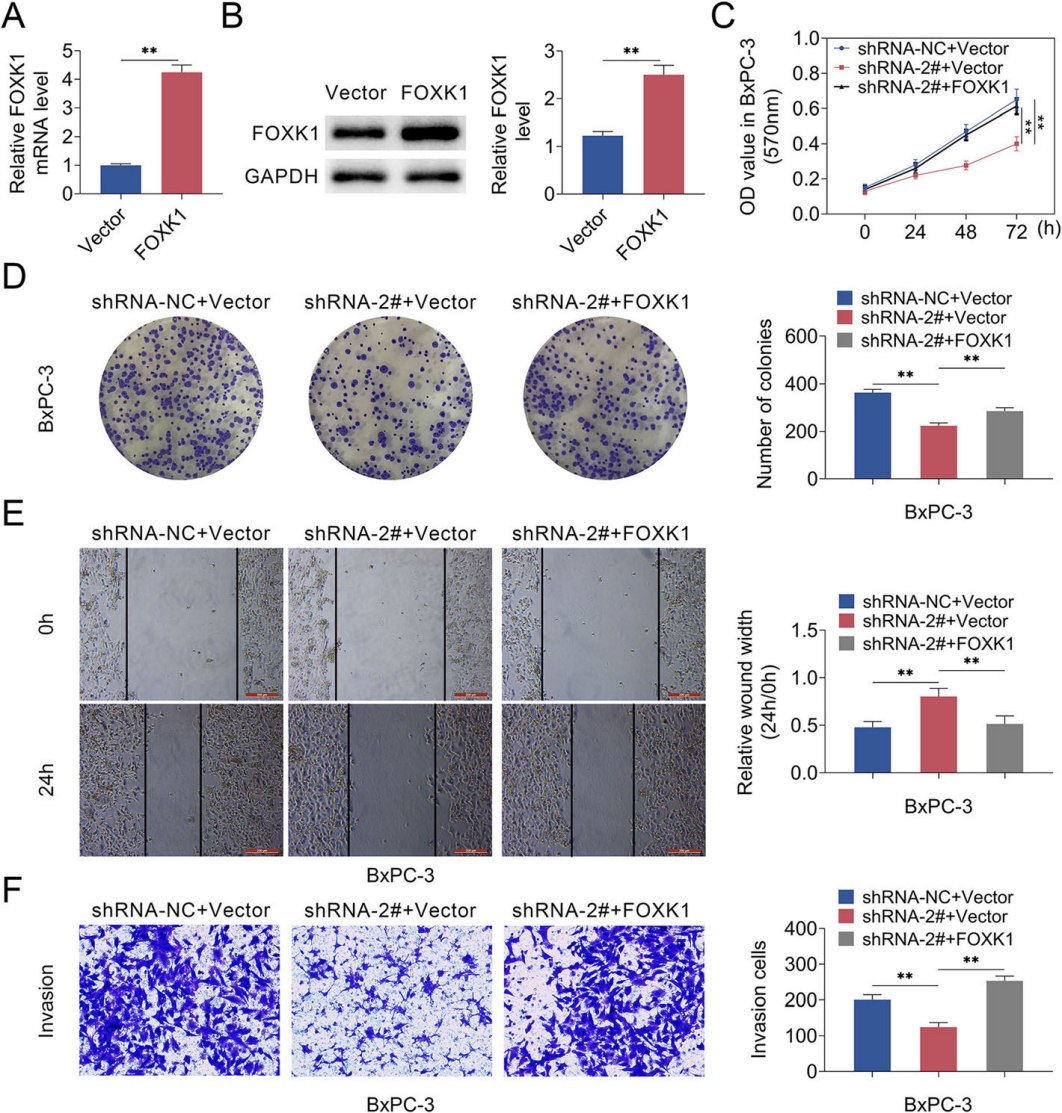
MCM3AP-AS1 facilitated cell growth and invasion in PC cells by FOXK1. **a** qRT-PCR and western blot analysis showed the FOXK1 level. **c** Cell proliferation was detected via MTT assay. **d** Clone formation assay was used to detect the clone formation ability. **e**-**f** Cell invasion and migration were testified using Transwell assay and wound healing assay. Scale bar, 50 μm. n = 3. (**, *p* < 0.01)

To investigate whether MCM3AP-AS1 facilitate growth and invasion by regulating FOXK1 expression, BxPC-3 cells were co-transfected with MCM3AP-AS1 silencing and FOXK1 overexpression constructs. Firstly, qRT-PCR and western blot analysis testified that the FOXK1 level was higher in FOXK1-overexpression PC cells (Fig. 6a and b). MCM3AP-AS1 silencing inhibited proliferation, which could be rescued in BxPC-3 cells co-expressing MCM3AP-AS1 silencing and FOXK1 overexpression (Fig. 6c). MCM3AP-AS1 silencing repressed cell formation, cell invasion as well as migration abilities. However, FOXK1 overexpression reversed the suppression of cell properties induced by MCM3AP-AS1 silencing (Fig. 6d-f). These data suggested that MCM3AP-AS1 facilitated cell growth and invasion in PC cells by FOXK1.

## Discussion

In current study, the MCM3AP-AS1 was elevated in PC tissues and cell models, which was found to promote cell growth and invasion in PC cells. MCM3AP-AS1 knockdown suppressed tumor growth in mice. Moreover, MCM3AP-AS1 was confirmed to sponge miR-138-5p, and FOXK1 was identified to bind with miR-138-5p. Further functional analysis showed that MCM3AP-AS1 promoted FOXK1 expression by targeting miR-138-5p, illustrating MCM3AP-AS1 could facilitate cell growth and invasion in PC cells by FOXK1.

Previously, MCM3AP-AS1 was reported to regulate cell processes by targeting miRNAs. For example, Zhang et al. discovered MCM3AP-AS1 stimulated cell metastasis in hepatocellular carcinoma through interacting with miR-455 (Zhang et al. 2019b). Liang et al. proved that MCM3AP-AS1 enhanced cell proliferation and invasion in papillary thyroid cancer by targeting miR-211-5p (Liang et al. 2019b). Similar to previous studies, we discovered that MCM3AP-AS1 was increased in PC patients and models. MCM3AP-AS1 overexpression promoted proliferation, invasion as well as migration, elevated PCNA, MMP2 as well as MMP9 protein levels and reduced p21 protein level. Conversely, MCM3AP-AS1 silencing showed the opposite effect. Moreover, MCM3AP-AS1 knockdown repressed tumor growth in mice. Notably, MCM3AP-AS1 could sponge miR-138-5p. The negative correlation was validated between MCM3AP-AS1 and miR-138-5p. Accumulating evidences have implied miR-138-5p took part in the suppression of cell migration and invasion in breast cancer (Zhao et al. 2019). Additionally, miR-138-5p overexpression was reported to reduce cell viability, migration as well as invasion in retinoblastoma (Wang et al. 2017). These findings suggested MCM3AP-AS1 might stimulate cell growth in PC by inhibiting miR-138-5p.

Besides, this study testified that miR-138-5p could directly target FOXK1. It is reported that FOXK1 level was higher in liver cancer cells than in normal human hepatic cell line, FOXK1 silencing was found to repress cell viability (Cui et al. 2018). Moreover, FOXK1 level was elevated in glioma tissue samples and cell lines, and FOXK1 was identified to enhance cell growth in glioma (Ji and Jiang 2018). Interestingly, we discovered that FOXK1 level was increased in PC tissues, suggesting that FOXK1 might be employed by PC cell to stimulate cell growth. Importantly, positive correlation between MCM3AP-AS1 and FOXK1 was found, whereas negative correlation between miR-138-5p and FOXK1 was also confirmed. MCM3AP-AS1 silencing led to decreased FOXK1 level, whereas FOXK1 level was reversed in BxPC-3 cells expressing MCM3AP-AS1-silencing and miR-138-5p inhibitor. Furthermore, we also demonstrated that MCM3AP-AS1 stimulated growth and invasion in PC cells by regulating FOXK1 expression. Notably, previous studies have discovered that lncRNAs participated in the progression of tumors by targeting miRNAs and regulating FOXK1 expression. For instance, Dong et al. discovered LINC02163 could augment growth in gastric cancer cells by miR-593-3p/FOXK1 axis (Dong et al. 2018). Lu et al. proved LINC01503 facilitated cell proliferation and invasion in colorectal cancer via miR-4492/FOXK1 axis (Lu et al. 2018). Consistent with the previous studies, our study revealed that MCM3AP-AS1 promoted PC cell growth and invasion by miR-138-5p/FOXK1 in vitro and in vivo, demonstrating the significant role of MCM3AP-AS1/miR-138-5p/FOXK1 axis in PC.

However, miR-138-5p was also predicted to bind with other targets, therefore MCM3AP-AS1 might be involved in PC in other signaling pathways. The detailed mechanisms of MCM3AP-AS1 underlying cell growth and invasion in PC should be further explored. The additional experiments will be conducted in the future.

## Conclusions

In conclusion, this study discovered MCM3AP-AS1 promoted cell proliferation, migration and invasion in PC through regulating miR-138-5p and FOXK1, providing insights into MCM3AP-AS1/miR-138-5p/FOXK1 axis as new candidates targeting PC therapeutics for drug design and development from bench to clinic.

## Data Availability

All data generated or analyzed during this study are included in this published article.

## Abbreviations

MCM3AP-AS1: MCM3AP antisense RNA 1
miR-138-5p: microRNA miR-138-5p
PC: Pancreatic cancer
